# Antibacterial and Antibiofilm Activity of Nanostructured Copper Films Prepared by Ionized Jet Deposition

**DOI:** 10.3390/antibiotics12010055

**Published:** 2022-12-29

**Authors:** Daniele Ghezzi, Enrico Sassoni, Marco Boi, Matteo Montesissa, Nicola Baldini, Gabriela Graziani, Martina Cappelletti

**Affiliations:** 1 BST Biomedical Science and Technologies and Nanobiotechnology Lab, IRCCS Istituto Ortopedico Rizzoli, Via di Barbiano 1/10, 40136 Bologna, Italy; 2 Department of Civil, Chemical, Environmental and Materials Engineering, University of Bologna, Via Terracini 28, 40131 Bologna, Italy; 3 Department of Biomedical and Neuromotor Sciences, University of Bologna, Via Massarenti 9, 40128 Bologna, Italy; 4 Department of Pharmacy and Biotechnology, University of Bologna, Via Irnerio 42, 40126 Bologna, Italy

**Keywords:** metal coatings, copper, antibacterial activity, antibiofilm, Ionized Jet Deposition, Calgary Biofilm Device

## Abstract

Metal coatings represent good strategies to functionalize surfaces/devices and limit bacterial contamination/colonization thanks to their pleiotropic activity and their ability to prevent the biofilm formation. Here, we investigated the antibacterial and antibiofilm capacity of copper coatings deposited through the Ionized Jet Deposition (IJD) on the Calgary Biofilm Device (CBD) against the growth of two gram-negative and two gram-positive pathogenic strains. Three areas (i.e., (+)Cu, (++)Cu, and (+++)Cu based on the metal amount) on the CBD were obtained, presenting nanostructured coatings with high surface homogeneity and increasing dimensions of aggregates from the CBD periphery to the centre. The coatings in (++)Cu and (+++)Cu were efficient against the planktonic growth of the four pathogens. This antibacterial effect decreased in (+)Cu but was still significant for most of the pathogens. The antibiofilm efficacy was significant for all the strains and on both coated and uncoated surfaces in (+++)Cu, whereas in (++)Cu the only biofilms forming on the coated surfaces were inhibited, suggesting that the decrease of the metal on the coatings was associated to a reduced metal ion release. In conclusion, this work demonstrates that Cu coatings deposited by IJD have antibacterial and antibiofilm activity against a broad range of pathogens indicating their possible application to functionalize biomedical devices.

## 1. Introduction

Nosocomial infections are caused by a variety of organisms, including bacteria, fungi, viruses, parasites, and other agents. Infections can be derived from exogenous or endogenous sources and are transferred by either direct or indirect contact between patients, healthcare workers, contaminated biomedical devices, and/or visitors [1]. A survey of hospital-acquired infections (HAI) in the United States in 2011 reported more than 700,000 reported cases, with 75,000 deaths associated with nosocomial infections [2]. Among these, biofilm-associated microbial infections are among the most severe complications in clinics. The biofilm is a structured community of microbial cells that are firmly attached to a surface and have unique metabolic and physiological attributes by secreting a matrix composed of a complex array of extracellular polymeric substances (EPS). The EPS acts as a barrier against the penetration of dangerous molecules and preserve the microbes from physical and chemical damages (e.g., UV radiations, desiccation, detergents, pH and temperature shifts) [3,4]. Within a biofilm, both horizontal gene transfer and intercellular communication (quorum sensing) are promoted between the microbial cells leading to possible transmissions of antibiotic resistance genes and increasing the development of multidrug resistant microbes [5].

The abuse of antibiotics treatments has led to the urgent need of alternative strategies in clinics [6,7,8,9,10]. The search for antimicrobials with pleiotropic activities is encouraged to prevent, or at least delay, the development of bacterial resistances, which, on the contrary, is a common and rapid phenomenon occurring when bacteria have long contact with a target-specific molecule. Metals are used as antimicrobials due to both their efficacy at low dosage and the hardness for microbes to develop resistances [11]. To address infections, the use of metal-based compounds is promising, as they have toxic effects against multiple molecular targets within the microbial cells compared to conventional organic antibiotics [12,13]. The functionalization of surfaces/devices with metals is a promising strategy as it provides antiadhesive and/or antimicrobial activities against a wide range of bacterial and fungal pathogens [14,15]. The toxic effect of metals was observed to be significantly higher towards prokaryotic cells compared to human cell lines, demonstrating biocompatibility of the coated materials [16,17,18]. The majority of the literature and of the solutions in the clinics in the past decades has been focusing on the use of silver in the form of coatings, cements, solutions, colloids, and nanoparticles [19,20,21]. However, different metals have different efficacy towards different strains, with some metals, such as zinc and copper, generally having higher efficacy against the gram-positive strains [22], including *S. aureus*, which is one of the main bacterial species responsible for orthopaedic infection [23]. In particular, copper appears very promising since it is already largely used for surfaces, including those in hospitals and healthcare facilities, where it is being increasingly used to replace non-antibacterial surfaces [19]. The biocide properties of copper have been known for centuries, and copper-based surfaces have been widely exploited for the prevention of microbial contamination. Its efficacy was demonstrated against pathogenic bacteria, fungi, and viruses [24,25,26,27,28], and it has been registered at the U.S. Environmental Protection Agency (EPA) as the first solid antimicrobial material [27]. At the same time, the literature data indicate that copper has scarce toxicity towards healthy cells [19,28,29] and that, in some cases, it can have a positive effect by enhancing angiogenesis and osteogenesis [30,31], both aspects being crucial for the application to implantable devices (such as orthopaedic prostheses) and for wound healing.

Transition metal copper is an essential chemical element for micro-organisms. It is directly involved in fundamental enzymatic reactions as proteins cofactor, like those included in the oxidative phosphorylation and photosynthesis [32]. Despite its physiological role in the bacterial cell, copper can have a toxic effect when present in excess in the surrounding environment [32]. Copper showed important inhibitory activities against various microbial species, including gram-negative and gram-positive reference pathogenic strains, Methicillin Resistant *Staphylococcus aureus* (MRSA), and Vancomycin Resistant Enterococci (VRE) strains [33,34]. The bactericidal effect of copper was demonstrated to proportionally increase with its concentration in the environment [35] and is mainly attributed to the release of ions, which affect the integrity of the membrane and/or the bacterial wall, generate intracellular oxidative stress, and are genotoxic, resulting in the death of micro-organisms [31,36]. One advantage of copper as a bactericidal agent is the low level of resistance among clinically relevant micro-organisms, whereas copper-resistant mechanisms were primarily found in environmental micro-organisms living in copper-rich niches, such as marine sediments and mines [37]. Despite its high and wide-spectrum antimicrobial activity and the rising interest over its use in biomedicine, copper coatings are still scarcely investigated for biomedical devices, and copper-coated devices are not yet available in clinics. However, studies on copper-doped calcium phosphates [38] and bioactive glass [30] nanocoatings showed promising results in terms of antimicrobial performance.

In this work, nanostructured copper films were deposited for the first time by Ionized Jet Deposition (IJD). The IJD is an innovative plasma-assisted technique that obtains nano-thick coatings, preserving the stoichiometry from the target to the coatings and providing both a nanostructured and highly specific surface to guarantee a sustained release of the antibacterial agent [17,39,40,41]. Depositions of antimicrobial films by IJD (based on metals and/or metal/ceramic composites) have been recently proposed by a research group [17,40,42], but copper-based compounds have not been tested yet. The validation procedure is also innovative as it is based on the use of the Calgary Biofilm Device (CBD), an optimized 96-well microtiter plate for biofilm studies that possess a coverlid with supports (pegs) entering the wells [43,44,45]. Copper coatings are deposited directly on the surface of the CBD for validation. In addition, IJD coats several substrates, including heat sensitive and porous ones, thus it can be used for surfaces, DPIs, fabrics, and implantable devices. Both the wells and the pegs were used as substrate to evaluate the inhibition of the copper coating and ions release on the planktonic growth and biofilm formation of two gram-positive and two gram-negative common human pathogenic bacterial strains.

## 2. Results

### 2.1. Composition and Morphology of the Copper Coatings

**Figure 1:**
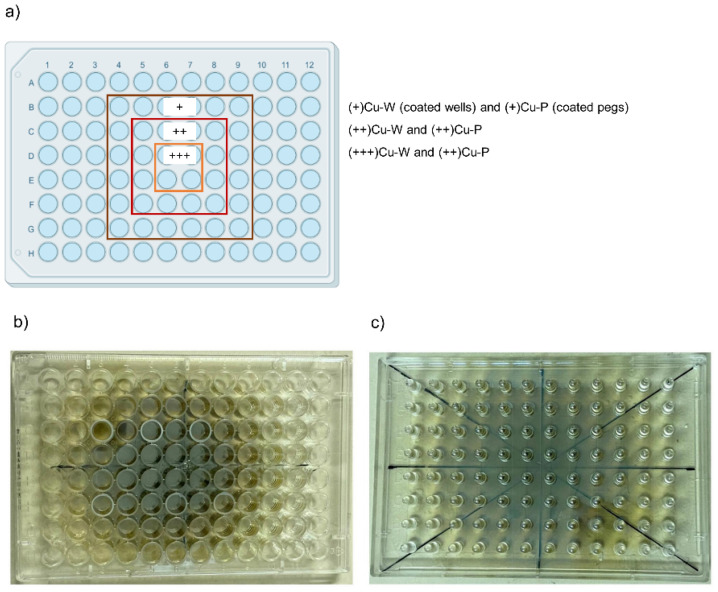
The three areas (**a**) on the Calgary Biofilm Device coated with copper on wells (**b**) and pegs (**c**). The boxes containing the indication (+)Cu, (++)Cu, and (+++)Cu correspond to the wells/pegs in the CBD areas that have coatings with the same concentrations, i.e., low Cu concentration for (+)Cu, medium Cu concentration for (++)Cu, and high Cu concentration for (+++)Cu.

The coatings were deposited onto the CBD without any pre- or post-deposition treatment. Because of the characteristics of the deposition procedure, where the amount of target material to be deposited depends on the distance to the center and the source of the plasma plume, the support is covered by the targeted metal (copper) in a concentric way, so that the highest concentration of the metal is in the four central wells and pegs, then the amount progressively decreases by moving towards the external perimeter of the support. Hence, we identified three main homogeneous areas in the CBD plate hereafter listed based on the amount of deposited copper (as depicted in Figure 1):

- Low metal amount [named (+)Cu], corresponding to the external area;
- Medium metal amount [(++)Cu] in the central area;
- High metal amount [(+++)Cu] in the inner area of the CBD.

**Figure 2:**
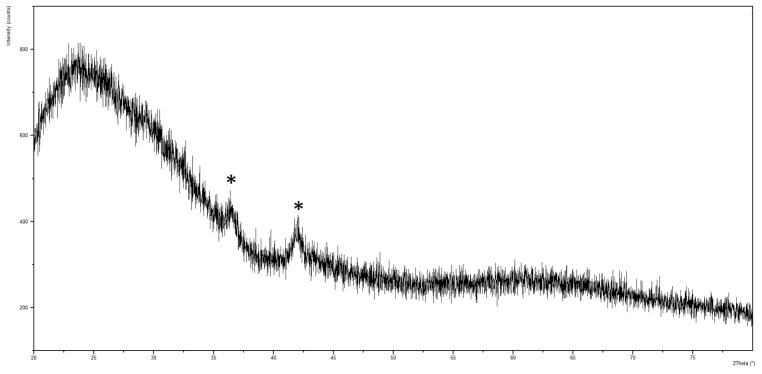
XRD spectrum of copper-based films. Peaks relative to copper oxide (Cu_2_O, ICDD 00-005-0667) are marked with *.

XRD shows that films are constituted by copper oxide (Cu_2_O, ICDD 00-005-0667, Figure 2). The presence of the only peaks at 2θ = 36.5 and 41.9 indicate a preferential orientation of the crystals along the (1 1 1) and (0 0 2) directions, respectively, as already found in the literature for copper-based films deposited by magnetron sputtering [46].

**Figure 3:**
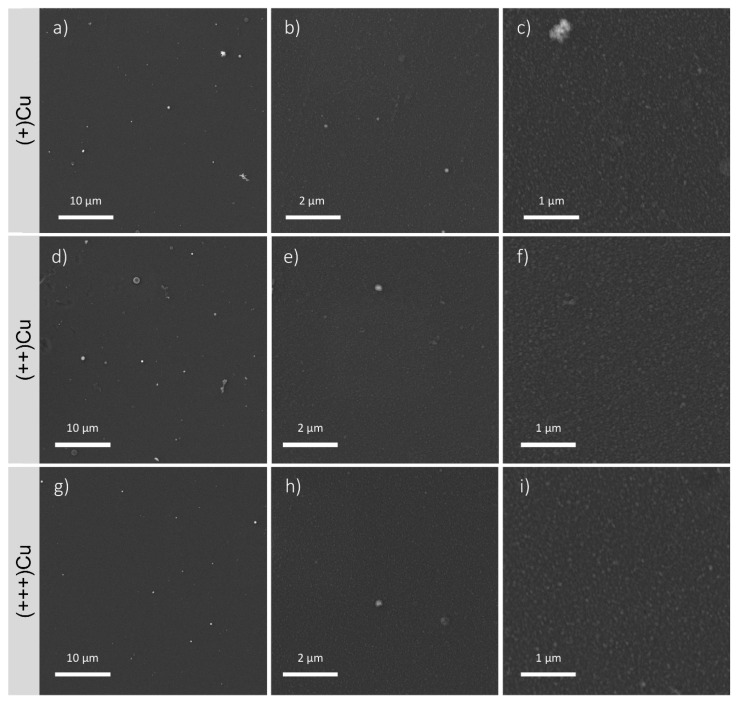
FEG-SEM images of the coatings in the different areas of the CBD at different magnifications (5000×, 20,000×, 50,000×): (**a**–**c**) (+)Cu, (**d**–**f**) (++)Cu, (**g**–**i**) (+++)Cu. In the Figures at higher magnifications (50,000×, (**c**,**f**,**i**)), the presence of homogeneous nanosized aggregates is clearly visible.

All films are nanostructured and show high surface homogeneity. Indeed, images at high magnification (50,000×, Figure 3c,f,i) show that the films are constituted by nanoscale aggregates with homogeneous dimensions. Images at lower magnification (5000× Figure 3a,d,g, and 20,000×, Figure 3b,e,h) show that the random aggregates of larger dimensions are present but infrequent and are mostly concentrated in (++)Cu. Results obtained by aggregates measuring by image J (Table 1) indicate an increasing trend in the aggregates’ dimensions, moving from the external to the inner areas of the CBD. Indeed, the maximum diameter progressively increases from (+)Cu to (++)Cu. The minimum diameter appears lower in the external area, but the presence of thinner aggregates, not detected by SEM, cannot be excluded.

As for the films in (+)Cu, due to thin thickness, the presence of the film is scarcely noticeable at lower magnifications, but it can be verified by scratching the film from the substrate (Supplementary Figure S1).

When coatings are deposited onto titanium alloy cylinders (Figure S2), they do not alter the substrate finishing at the micro- and macro-scale, as recommended for orthopaedic implants where substrate surface finishing is designed to maximize primary stability. Because the coatings have nanoscale thickness, their presence is not clearly visible. Indeed, while the aggregates of smaller dimensions can be recognized on the implants surface, the larger ones are concealed by the substrate surface finishing. Minimum diameters of 19 nm are measured for the zone (+)Cu and of 28 nm for (++)Cu and (+++)Cu, comparable to those observed for deposition onto the CBD. This indicates that this characteristic of the coating is not significantly affected by deposition substrate. Although the coatings cannot be clearly detected by SEM, their presence can be confirmed by EDS. Indeed, EDS indicates a copper presence of about 20% in (+)Cu and about 35–40% in (++)Cu and (+++)Cu. For the latter, the concentration of copper is essentially the same, indicating how moving from the two areas results in aggregates coarsening rather than a much higher amount of deposited copper. This behaviour had been already observed for silver coatings deposited by IJD (data in press), where areas with medium and high metal concentration showed similar film thickness, roughness, and Ag-ion release, but different aggregates’ morphology, ultimately leading to different antimicrobial behaviour. In addition, the higher presence of large aggregates might influence the overall amount of copper.

### 2.2. Evaluation of Antibacterial and Antibiofilm Properties on the Calgary Biofilm Device

**Figure 4:**
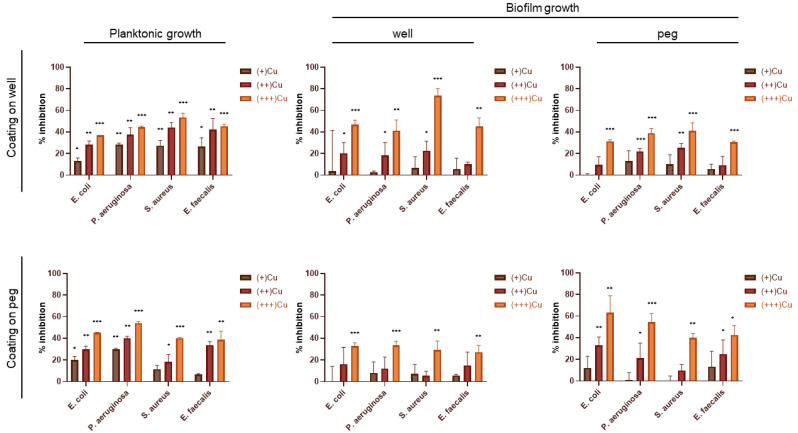
Antibacterial and antibiofilm activities exerted by Cu coatings against the four bacterial pathogens *E. coli*, *P. aeruginosa*, *S. aureus*, and *E. faecalis*. The results are reported as the mean of the percentage of bacterial and biofilm inhibition ± standard deviation. Statistical analysis was performed compared to control experiments (100% of growth) using one-way ANOVA test. Differences were considered significant for *p* < 0.05 (* *p* < 0.05, ** *p* < 0.01, *** *p* < 0.001).

The antibacterial and antibiofilm capacities of the Cu coatings against the two gram-negative *E. coli* and *P. aeruginosa* and the two gram-positive *S. aureus* and *E. faecalis* strains were evaluated in terms of (i) inhibition of the bacterial growth in planktonic form (suspended cells in (+/++/+++)Cu-W and (+/++/+++)Cu-P wells), (ii) inhibition of the biofilm formation on the coated support (cells attached as biofilms on wells in (+/++/+++)Cu-W or on pegs in (+/++/+++)Cu-P), and (iii) inhibition of the biofilm formation on the uncoated support (cells attached as biofilms on wells in (+/++/+++)Cu-P or pegs in (+/++/+++)Cu-W) (Figure 4).

For all the tested strains, the antibacterial effect of the Cu coating on wells against planktonic cells’ growth was significant for all the bacterial strains and with all the metal concentrations tested, although the growth inhibition levels decreased going from (+++)Cu-W to (+)Cu-W. The strongest effect was shown by coatings in (+++)Cu-W against the planktonic cells of *S. aureus* (50% of growth inhibition compared to the control), followed by *P. aeruginosa* and *E. faecalis* (43–45% of growth inhibition), and *E. coli* (36% of inhibition). By statistically comparing the planktonic inhibitory effect exerted by these coatings on each bacterial strain, the only significant difference was found between *S. aureus* and *E. coli* (Table S1). Cu coatings on pegs also had significant antibacterial effect against all the bacterial strains but only in (+++)Cu-P and (++)Cu-P areas. The strongest antibacterial effect of coatings in (+++)Cu-P was observed against *P. aeruginosa* (54% of growth inhibition). This inhibitory effect was statistically significant compared to the other three bacterial strains tested (Table S1). The coatings in (+)Cu-P showed significant inhibitory activity against both *P. aeruginosa* and *E. coli*, whereas the inhibition of *S. aureus* and *E. faecalis* was not statistically significant (Figure 4).

The results of the biofilm formation inhibition on the coated support, i.e., the effect of the direct contact of the Cu coating with the bacterial cells, showed a metal concentration-dependent action as observed in the planktonic cells’ growth inhibition. In particular, the antibacterial effect of copper against the biofilm formation on the coated support was significant for all the bacterial strains in (+++)Cu-W and (+++)Cu-P. The highest effect in terms of biofilm formation was observed with the coatings in (+++)Cu-W against *S. aureus* (around 74% compared to the control). This antibacterial activity was significantly higher only compared to *P. aeruginosa* (Table S1). In the case of (+++)Cu-P, the biofilm formation on the coatings showed around 63% inhibition of *E. coli* and 54% of *P. aeruginosa*, and 40% for *S. aureus* and *E. faecalis*. (++)Cu-W and (++)Cu-P still retained significant antibacterial activity against three out of four bacterial strains tested, while (+)Cu-W and (+)Cu-P did not show any significant activity (Figure 4). The different activity between Cu-W and Cu-P might be due to differences between wells and pegs as deposition substrates and/or their different positioning as surfaces for biofilm attachment [47].

The antibiofilm effect of the Cu ions’ release alone, i.e., without considering the direct contact cell-coating, was evaluated by measuring the biofilm formation on the uncoated well or peg. The inhibition of the biofilm formation on the uncoated peg in (+++)Cu-W ranged between 30% and 40% for all bacterial strains with no significant differences among them (Table S1). The coatings in (++)Cu-W had a significant antibiofilm activity on the uncoated pegs for *S. aureus* and *P. aeruginosa* (25% and 22% of biofilm inhibition, respectively), whereas in (+)Cu-W no significant inhibition was observed for any of the tested strains. The effect of coatings in (+++)Cu-P was significant for all bacterial strains (27–33% of biofilm inhibition) with no differences among them. No statistically significant inhibition of the biofilm formation was observed in the (++)Cu-P and (+)Cu-P areas for any of the tested bacterial strains (Figure 4).

## 3. Discussion

In this study we evaluated the effect of Cu coatings deposited for the first time with the Ionized Jet Deposition technology, which is suitable to produce nanostructured thin coatings with submicrometric thickness and prolonged ion release, as previously demonstrated for antibacterial silver coatings [17,39,40,41]. By applying the IJD technology to the Calgary Biofilm Device, a 96-wells multiplate optimized for the investigation of the microbial biofilm formation and equipped with wells-fitting pegs on the plate lid [43,44,45], we obtained nanostructured coatings composed by spherical aggregates having diameters ranging from 15 to 115 nm and whose characteristics depend on the position with respect to the plasma plume and, hence, its angle of incidence. The coatings can be deposited onto a variety of substrates, including Titanium-Aluminium-Vanadium prostheses, for perspective applications in orthopaedics. In the latter case, thanks to the nanoscale thickness and the conformal growth of the coatings (already observed for silver films [48]), they do not alter the microscale surface finishing of the implant. During the deposition phase, three areas were formed in the CBD characterized by coatings with three different Cu concentrations, i.e., low (+)Cu, medium (++)Cu, and high (+++)Cu. The coatings were deposited on either pegs or wells to determine the effect of direct contact on biofilms formed on coated wells or pegs, or the effect of metal ion release (from coatings) on biofilms formed on uncoated pegs or wells.

The antibacterial activity of the IJD-deposited Cu coatings was tested against two gram-negative and two gram-positive bacterial strains belonging a panel of the bacterial species indicated in the EPA protocols and/or associated to hospital-acquired infections [2,49,50], i.e., *Escherichia coli*, *Pseudomonas aeruginosa*, *Staphylococcus aureus*, and *Enterococcus faecalis* species. Strains belonging to these four bacterial species are known to cause severe clinical infections thanks to their ability to form biofilm both on the human tissues and on the materials applied for the prostheses [51]. The antibacterial activity was shown to be dependent on the characteristics of the copper films, which, in turn, was associated with the three CBD areas that were hit by the plasma plume with different angles. This behaviour was observed both on the planktonic cells growth and on the biofilm formation for all the tested bacterial strains. Several cellular mechanisms are thought to be involved in the antibacterial activities of copper, although some of them are still not fully elucidated [52]. The production of Reactive Oxygen Species (ROS) is considered one of the most common outcomes of the interaction between the bacteria and exceeded amount of copper. This is mainly due to the boundary of copper with oxygen and the consecutive formation of peroxide radicals [52,53]. However, contact-killing studies on dry copper alloy surfaces assessed that the first effect of copper on bacteria is represented by the peroxidation of unsaturated fatty acids leading to the disruption of the cellular structure and the failing of the membrane integrity [54,55,56].

The antibacterial effect in terms of inhibition of the planktonic growth was significant in all the three CBD areas and for all the tested strains under analysis areas with the highest concentrations of copper, i.e., (+++)Cu-W/P and (++)Cu-W/P. Generally, we observed an antibacterial activity of copper coatings that was strictly dependent on the metal concentration as previously stated [36]. This is probably due to the fact that decreasing the Cu concentration in the coatings reduces the amount of copper ions released that might be not enough to destabilize the bacterial physiological conditions and/or overcome the bacterial resistance mechanisms [32,35]. In terms of antibacterial efficiency, we identified a maximal planktonic growth inhibition (in (+++)Cu-W/(+++)Cu-P areas) of 50%/40% for *S. aureus*, 44%/54% for *P. aeruginosa*, 45%/39% for *E. faecalis*, and 36%/45% for *E. coli*. The results obtained for *E. coli* were in line with those described for an *E. coli* ATCC strain exposed to copper nanoparticles supplied to the medium at concentration 20–80 µg Cu^0^/mL, showing 25–50% of growth inhibition over a period of 48 h [52]. Previous studies showed that copper nanoparticle coatings exerted an antibacterial activity against a wide range of bacteria, even sporulating ones like *Clostridium difficile*, food-borne pathogen *Escherichia coli* O157, and clinical isolates of *Klebsiella pneumoniae*, *Acinetobacter baumannii*, and *Pseudomonas aeruginosa* species [35,36,57,58]. On the other hand, the inhibition of biofilm formation was generally not significant (compared to the control), especially in the (+)Cu-W and (+)Cu-P areas. The different behaviour towards copper between the two modes of growth could be associated with the higher tolerance generally known for the cells growing as biofilms rather than in planktonic form. Tolerance mechanisms associated with biofilm growth can be mediated by the characteristics of the EPS produced that can limit the contact between antimicrobial compounds and the cells, as well as metabolic and physiology heterogeneity that can give rise to subpopulations that are resistant/tolerant to antimicrobials because they are metabolically inactive and/or have persisting states [59]. EPS synthesis and binding properties have been previously associated to copper resistance/tolerance mechanisms in *P. aeruginosa* strains [60]. The biofilm formation on the uncoated wells and uncoated pegs in (+)Cu-W and (+)Cu-P was not significantly inhibited with all of the four bacterial strains under analysis. The inhibitory effect on biofilm growth on these uncoated supports could be associated with the ions released from the coated pegs and wells, respectively. Therefore, the antibacterial effect of copper on the biofilm formation appears to be higher when the cells are directly in contact with the coating. Accordingly, previous works have noticed a higher performance of copper compared to silver, which is the most used metal for coatings applied to clinics [61,62], in the case of the direct contact with the metal coating [63,64]. On the contrary, silver was shown to be more efficient compared to copper in terms of the ions release [65]. This is probably due to the copper toxicity that is strictly related to the local concentration that the bacterial cells perceive; indeed, differently from silver, copper is an essential element for the bacterial cell and, at physiological concentrations, copper ions are needed as cofactors in redox enzymatic reactions involved in many fundamental cellular metabolisms [32,36]. Accordingly, previous studies have demonstrated that when the copper concentration overcame specific threshold values, the antibacterial activity of the metal against *E. coli* and *S. aureus* drastically increased [66].

In our work, we found that the coatings characterized by high Cu concentrations were active against both gram-negative and gram-positive bacteria, i.e., the coatings in (++)Cu were active against planktonic cells and those in (+++)Cu were active against both planktonic and biofilm cells. The differences that we observed in terms of efficiency against different bacterial strains with the coatings in (++)Cu (for biofilm growth) and in (+)Cu (for planktonic growth) might be due to distinct EPS synthesis and composition that can be associated with specific metal sequestration capacities [67] and a strain-specific metal toxicity response that are independent of the gram staining categorization [32,67]. In this regard, various copper-resistance/adaptive strategies have been described in different gram-positive and gram-negative bacteria that might explain the differences we have observed among the strains tested in our study. If on the one hand the structural differences between gram-positive and gram-negative bacteria can contribute to different cell susceptibility and metal binding, on the other hand, mechanisms of copper resistance/tolerance associated to intracellular components exist in bacteria and are i) cellular export of copper (Cu transport from the cytoplasm into the periplasmic space in gram-negative strains, or into the extracellular environment in gram-positive strains), ii) copper sequestration by metallothioneins and siderophores, and iii) oxidation of Cu(I) into the less toxic Cu(II) ion [32].

## 4. Materials and Methods

### 4.1. Preparation of the Copper Coatings onto the Calgary Biofilm Device

All films were manufactured by deposition of copper, by the Ionized Jet Deposition (Noivion srl, Rovereto, Italy), a plasma-assisted technique where a solid target material, either a ceramic or a metal (here, copper target, 99.999% purity, 2.00″ diameter, 0.250″ thickness, ±0.010 Kurt J. Lesker, Jefferson Hills, PA, USA) is ablated by a pulsed electron beam (100 ns of high energy, (10 J) electrons with high-density (109 W cm^−2^) power) and deposited onto a substrate. In IJD, the coatings are obtained by the ionization and subsequent deposition of the solid target material, so neither wet reactions nor liquid precursors are used.

To ensure coatings’ uniformity, copper targets were kept rotating inside the deposition chamber, and the first 5 min of deposition were performed on a shutter to clean the target surface from possible impurities. The deposition is performed in high vacuum and under a controlled flow of oxygen. The vacuum chamber is evacuated down to a pressure of 1.0 × 10^−7^ mbar by an EXT255H turbomolecular pump (Edwards, Crawley, UK) and then raised to 3 × 10^−4^ mbar by a controlled flow of oxygen, having a 99.999% purity level.

Deposition parameters are selected based on preliminary tests, aimed at achieving suitable deposition rate and coatings’ homogeneity. Based on these results, target-substrate distance, electron beam frequency, and working voltage are set to 8 cm, 22 Hz, and 7 kV, respectively. In IJD, coatings’ characteristics depend on the duration of the deposition and on the distance of the plasma plume, so either increasing deposition time, or increasing proximity to the plume centre can be used to obtain coatings of different thickness. Here, deposition time has been fixed to 30 min, and coatings’ characteristics were varied by varying the position in the deposition chamber. To do so, the Calgary Biofilm Devices (Innovotech Inc., Edmonton, AB, Canada) have been used directly as a deposition substrate selecting three uniform areas (having the same distance and angle with respect to the plasma plume), having different film thickness and copper concentration in the well. Composition (EDS Bruker probe coupled with a field emission gun scanning electron microscope) and morphology (FEG-SEM Tescan Mira3, CZ, working distance = 10 mm, voltage = 10 kV) of the films in the three areas were investigated, and accurate microbiological analyses were performed to fully characterize the antimicrobial performance of the films, depending on their characteristics.

To show possible application to real biomedical substrates, titanium alloy disks were also used as support for Cu coatings. To this aim, micro-rough medical grade titanium alloy disks were also selected (Grade 23 Titanium 6Al-4V ELI alloy, 5 mm of thickness, 5 mm of diameter, Citieffe S.r.l., surface roughness (Ra) 5 μm) to simulate coating of orthopaedic prostheses. Here, the dimensions of the specimens are only selected based on easiness of characterization, while surface finishing and roughness are specifically mimicking those of orthopaedic prostheses. The cylinders were placed in the deposition chamber so as to correspond to each of the three different zones in the CBD.

### 4.2. Characterization of the Copper Coatings

The coatings’ morphological characteristics were analysed by evaluating their morphology and composition by Field Emission Gun Scanning Electron Microscopy (FEG-SEM, Tescan Mira3, CZ, working distance = 10 mm, voltage = 5 kV) coupled with EDS (Energy Dispersive X-ray Spectrometry system—EDS, INCA Energy 200, Oxford Instruments, Abingdon, UK). To avoid cutting of the CBD, which could cause damage/alterations to the coatings, plastic coverslips of the same material and geometry of the wells were inserted in the different wells of the CBD and extracted for microscopy analyses. Coatings’ uniformity and surface texture associated to each area were then assessed by FEG-SEM. Starting from images at 50 kX magnification, the maximum and minimum size of the aggregates constituting the coatings were measured by imageJ. Coatings’ composition was analysed for samples (+++)Cu by XRD, using a Malvern PANalytical Empyrean series III instrument (40 kV and 30 mA, 2θ range = 20–80, step size = 0.01, time per step = 30 s). For XRD analyses, coatings were deposited on 2 × 2 mm^2^ glass slides so that no bands deriving from the substrate could conceal those of the coating. Then, analyses were performed on coated titanium alloy cylinders, to verify absence of modification to substrate finishing. EDS was used on the cylinders to verify coatings’ presence and to indirectly quantify the coatings deposited on the cylinders, which correlates with thickness.

### 4.3. Antibacterial and Antibiofilm Assays

Bacteria included in this study included four human pathogenic strains, i.e., the gram-negative *Escherichia coli* ATCC 8739 and *Pseudomonas aeruginosa* PAO1, and the gram-positive *Staphylococcus aureus* ATCC 6538P and *Enterococcus faecalis* DP1122. For each strain, a single colony grown on Tryptic Soy Agar (TSA: casein peptone 15 g L^−1^, soya peptone 5 g L^−1^, sodium chloride 5 g L^−1^, agar 15 g L^−1^, pH 7.3. Sigma-Aldrich) was inoculated in 5 mL of Tryptic Soy Broth (TSA composition without agar. Sigma-Aldrich, (St. Louis, MO, USA)) and incubated under agitation at 37 °C overnight. All the cultures were diluted to reach an optical density of 0.03 measured at 600 nm (OD_600_). A volume of 150 μL of the obtained suspensions was transferred into the CBDs, which were incubated at 37 °C with gentle shaking (100 rpm) for 48 h in a humid environment. After incubation, the bacterial suspensions were transferred into a clean 96-multiwell to measure the absorbance (OD_600_) as an indication of the bacterial planktonic growth. The biofilm formation on the wells and on the pegs of the CBD was quantified through crystal violet (CV, Sigma-Aldrich, purity > 90%) staining. Briefly, both wells and pegs were washed twice with saline solution (NaCl 0.85% *w/v*, Sigma-Aldrich) to remove the non-adherent bacterial cells. The biofilms were fixed with 99% *v/v* ethanol (Supelco (St. Louis, MO, USA), 99% purity) for 10 min at room temperature and then stained with CV (0.2% w/v dissolved in water) for 10 min at room temperature. After removal of unbound CV with sterile water, acetic acid 33% *v/v* (Sigma-Aldrich, 99% purity) was added to solubilize the stained biofilms, which were quantified by measuring the absorbance at 595 nm. Controls were performed on CBD under the same growth conditions using uncoated wells and pegs. The background staining was corrected by subtracting the mean value for CV bound to negative controls (CBD wells with the growth medium but without the bacterial inoculum).

The growth inhibition (% inhibition in Figure 4) was calculated as follows: % inhibition = (1 − T/C) × 100, where T and C are cell density (measured as OD_600_ for planktonic and OD_595_ for biofilm) in the target experimental samples (inoculated CBD wells with coatings) and control samples (inoculated CBD wells without coatings), respectively.

All the microbiological results are reported as average ± standard deviation (SD) (*n* > 3). Statistical analysis was performed using one-way ANOVA test [68] using GraphPad Prism version 8.0.1 for Windows (GraphPad Software, San Diego, CA, USA ). Differences were considered significant when *p* < 0.05. A mixed model ANOVA with post hoc Tukey adjustment [69] was used to evaluate differences among bacterial strains.

## 5. Conclusions

This work describes the antibacterial and antibiofilm capacities of newly developed copper coatings prepared by Ionized Jet Deposition. By high-throughput screening assay Calgary Biofilm Device technology we observed that the obtained copper coatings successfully inhibited the growth of planktonic and biofilm cells of four bacterial pathogens associated with hospital-associated infections, in a metal concentration dependent way and without gram specificity. These results indicate the promising applications of these newly developed coatings for the functionalization of surfaces and biomedical devices. For the application to implantable devices, a thorough evaluation of biocompatibility, in terms of absence of cytotoxicity and interference with host cells’ proliferation and differentiation, is mandatory and is currently under evaluation.

Because IJD is a versatile technique, making feasible the deposition onto a variety of substrates, the proposed films have perspective applications in several biomedical fields, for antibacterial surfaces, DPIs, surgical equipment, and prostheses (including custom-made ones). The coatings can find an application into many diverse fields where antimicrobial coatings are required, including agriculture, building materials, water remediation, and air filters.

## Figures and Tables

**Table 1:** Aggregates’ minimum (Dmin) and maximum (Dmax) diameter.

CBD Area	Dmin (nm)	Dmax (nm)
(+)Cu	14	85
(++)Cu	22	96
(+++)Cu	22	112

## Data Availability

All data generated or analyzed during this study are included in this published article.

## Supplementary Materials

The following supporting information can be downloaded at: https://www.mdpi.com/article/10.3390/antibiotics12010055/s1, Figure S1: Thin films in (+)Cu. In white circles, the films have been purposely scratched from the substrates to make them more visible. Figure S2: FEG-SEM images of Cu coatings deposited at the three different concentrations tested: (a–c) (+)Cu, (e–g) (++)Cu, and (i–k) (+++)Cu. In (d,h,l), Energy Dispersive X-ray Spectroscopy (EDS) acquisitions of Titanium-Aluminium-Vanadium alloys corresponding to (+)Cu, (++)Cu, and (+++)Cu are reported, respectively. Table S1: Post-Hoc Tukey HSD test from ANOVA analysis for differences among bacterial strains.
