# Supplementary material for: Antibacterial and Antibiofilm Activity of Nanostructured Copper Films Prepared by Ionized Jet Deposition
Source: Antibiotics (Basel). 2022 Dec 29;12(1):55. doi: 10.3390/antibiotics12010055 (PMC9854604; doi:10.3390/antibiotics12010055)
Supplement: Supplementary file 1 [file antibiotics-12-00055-s001.zip › antibiotics-2106996-supplementary.pdf]

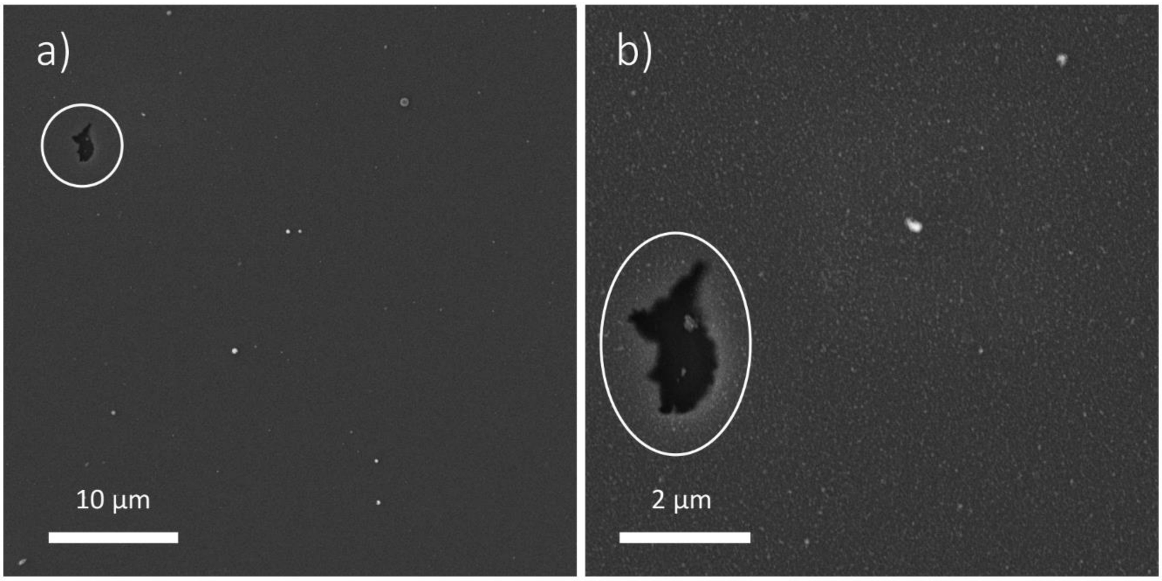

Figure S1: (**a**,**b**) Thin films in (+)Cu. In white circles, the films have been purposely scratched from the substrates to make them more visible.

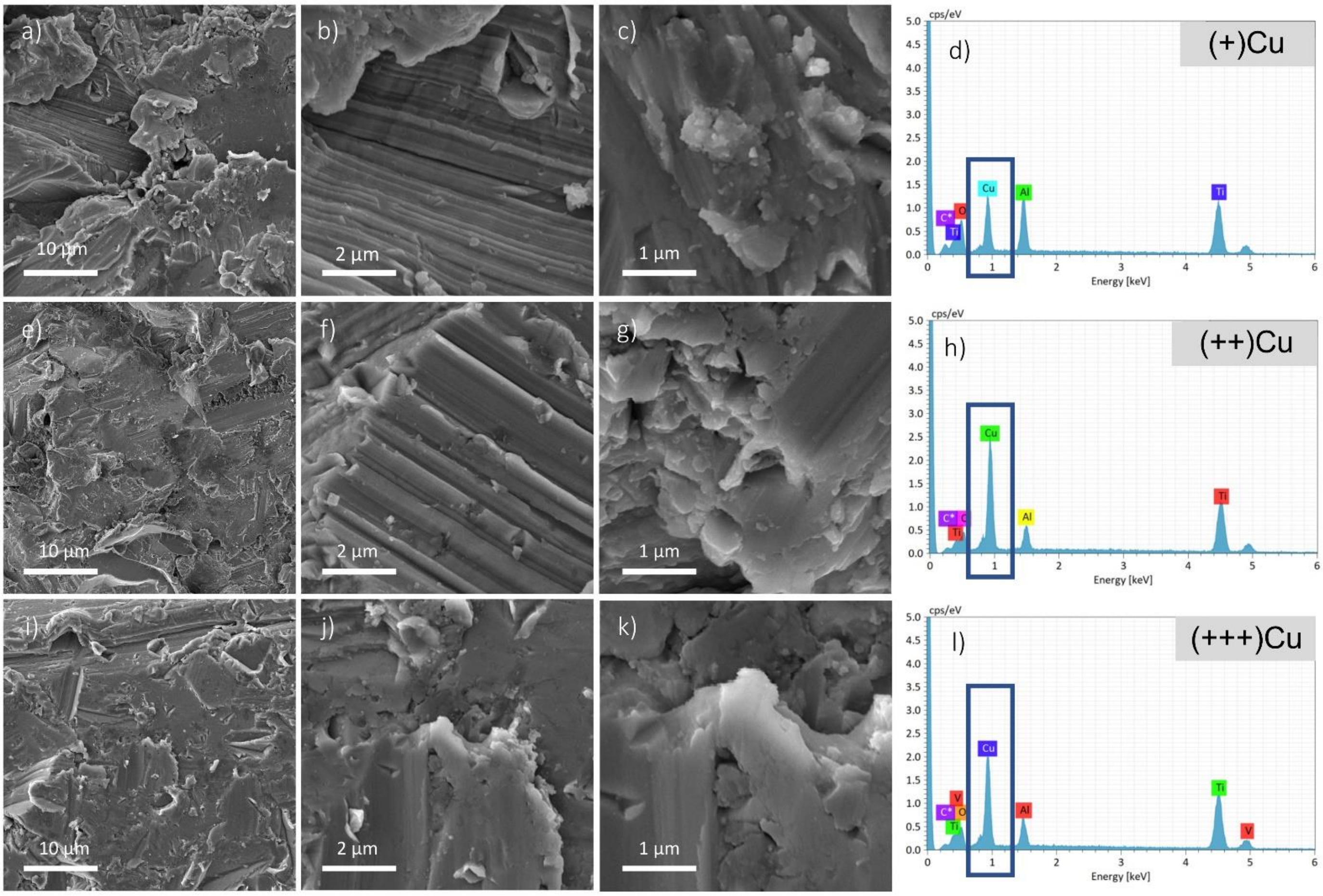

Figure S2: FEG-SEM images of Cu coatings deposited at the three different concentrations tested: (a–c) (+)Cu, (e–g) (++)Cu, i-k) (+++)Cu. In d, h, and l, Energy Dispersive X-ray Spectroscopy (EDS) acquisitions of aluminium-titanium vanadium alloys corresponding to (+)Cu, (++)Cu, and (+++)Cu are reported, respectively.

Table S1: *Post-Hoc* Tukey HSD test from ANOVA analysis for differences among bacterial strains.

Coated wells – Planktonic growth

Tukey's multiple comparisons test	Mean Diff	95.00% CI of diff	Significant?	Summary	Adjusted P Value
**+(Cu)**					
E. coli vs. P. aeruginosa	-15.37	-26.66 to -4.088	Yes	**	0.0050
E. coli vs. S. aureus	-14.43	-25.71 to -3.142	Yes	**	0.0087
E. coli vs. E. faecalis	-13.62	-24.90 to -2.331	Yes	*	0.0139
P. aeruginosa vs. S. aureus	0.9461	-10.34 to 12.23	No	ns	0.9955
P. aeruginosa vs. E. faecalis	1.757	-9.527 to 13.04	No	ns	0.9728
S. aureus vs. E. faecalis	0.8111	-10.47 to 12.10	No	ns	0.9972
**++(Cu)**					
E. coli vs. P. aeruginosa	-9.322	-20.61 to 1.962	No	ns	0.1312
E. coli vs. S. aureus	-16.12	-27.41 to -4.839	Yes	**	0.0032
E. coli vs. E. faecalis	-14.06	-25.34 to -2.775	Yes	*	0.0108
P. aeruginosa vs. S. aureus	-6.801	-18.09 to 4.483	No	ns	0.3645
P. aeruginosa vs. E. faecalis	-4.737	-16.02 to 6.547	No	ns	0.6582
S. aureus vs. E. faecalis	2.064	-9.220 to 13.35	No	ns	0.9572
**+++(Cu)**					
E. coli vs. P. aeruginosa	-7.476	-18.76 to 3.808	No	ns	0.2854
E. coli vs. S. aureus	-16.58	-27.86 to -5.296	Yes	**	0.0024
E. coli vs. E. faecalis	-8.394	-19.68 to 2.890	No	ns	0.1976
P. aeruginosa vs. S. aureus	-9.104	-20.39 to 2.180	No	ns	0.1449
P. aeruginosa vs. E. faecalis	-0.9175	-12.20 to 10.37	No	ns	0.9959
S. aureus vs. E. faecalis	8.186	-3.098 to 19.47	No	ns	0.2154

Coated wells – Biofilm on wells

Tukey's multiple comparisons test	Mean Diff	95.00% CI of diff	Significant?	Summary	Adjusted P Value
**+(Cu)**					
E. coli vs. P. aeruginosa	1.049	-29.28 to 31.38	No	ns	0.9997
E. coli vs. S. aureus	-2.982	-33.31 to 27.35	No	ns	0.9928
E. coli vs. E. faecalis	-1.954	-32.28 to 28.38	No	ns	0.9979
P. aeruginosa vs. S. aureus	-4.031	-34.36 to 26.30	No	ns	0.9827
P. aeruginosa vs. E. faecalis	-3.002	-33.33 to 27.33	No	ns	0.9927
S. aureus vs. E. faecalis	1.029	-29.30 to 31.36	No	ns	0.9997
**++(Cu)**					
E. coli vs. P. aeruginosa	1.891	-28.44 to 32.22	No	ns	0.9981
E. coli vs. S. aureus	-2.318	-32.65 to 28.01	No	ns	0.9966
E. coli vs. E. faecalis	10.20	-20.13 to 40.53	No	ns	0.7905
P. aeruginosa vs. S. aureus	-4.210	-34.54 to 26.12	No	ns	0.9805
P. aeruginosa vs. E. faecalis	8.305	-22.02 to 38.63	No	ns	0.8735
S. aureus vs. E. faecalis	12.52	-17.81 to 42.84	No	ns	0.6701
**+++(Cu)**					
E. coli vs. P. aeruginosa	5.281	-25.05 to 35.61	No	ns	0.9627
E. coli vs. S. aureus	-27.00	-57.33 to 3.327	No	ns	0.0933
E. coli vs. E. faecalis	1.672	-28.66 to 32.00	No	ns	0.9987
P. aeruginosa vs. S. aureus	-32.28	-62.61 to -1.954	Yes	*	0.0340
P. aeruginosa vs. E. faecalis	-3.609	-33.94 to 26.72	No	ns	0.9875
S. aureus vs. E. faecalis	28.67	-1.655 to 59.00	No	ns	0.0686

Coated wells – Biofilm on pegs

Tukey's multiple comparisons test	Mean Diff	95.00% CI of diff	Significant?	Summary	Adjusted P Value
**+(Cu)**					
E. coli vs. P. aeruginosa	-12.98	-26.38 to 0.4269	No	ns	0.0602
E. coli vs. S. aureus	-10.00	-23.40 to 3.403	No	ns	0.1955
E. coli vs. E. faecalis	-5.801	-19.20 to 7.602	No	ns	0.6365
P. aeruginosa vs. S. aureus	2.976	-10.43 to 16.38	No	ns	0.9271
P. aeruginosa vs. E. faecalis	7.176	-6.227 to 20.58	No	ns	0.4664
S. aureus vs. E. faecalis	4.199	-9.204 to 17.60	No	ns	0.8230
**++(Cu)**					
E. coli vs. P. aeruginosa	-12.69	-26.09 to 0.7124	No	ns	0.0680
E. coli vs. S. aureus	-15.65	-29.06 to -2.252	Yes	*	0.0178
E. coli vs. E. faecalis	0.2041	-13.20 to 13.61	No	ns	>0.9999
P. aeruginosa vs. S. aureus	-2.964	-16.37 to 10.44	No	ns	0.9279
P. aeruginosa vs. E. faecalis	12.89	-0.5084 to 26.30	No	ns	0.0623
S. aureus vs. E. faecalis	15.86	2.456 to 29.26	Yes	*	0.0162
**+++(Cu)**					
E. coli vs. P. aeruginosa	-7.386	-20.79 to 6.017	No	ns	0.4416
E. coli vs. S. aureus	-9.497	-22.90 to 3.906	No	ns	0.2329
E. coli vs. E. faecalis	0.9389	-12.46 to 14.34	No	ns	0.9974
P. aeruginosa vs. S. aureus	-2.111	-15.51 to 11.29	No	ns	0.9719
P. aeruginosa vs. E. faecalis	8.325	-5.078 to 21.73	No	ns	0.3389
S. aureus vs. E. faecalis	10.44	-2.967 to 23.84	No	ns	0.1670

Coated pegs – Planktonic growth

Tukey's multiple comparisons test	Mean Diff	95.00% CI of diff	Significant?	Summary	Adjusted P Value
**+(Cu)**					
E. coli vs. P. aeruginosa	-10.00	-18.28 to -1.720	Yes	*	0.0138
E. coli vs. S. aureus	8.363	0.08260 to 16.64	Yes	*	0.0471
E. coli vs. E. faecalis	13.51	5.226 to 21.79	Yes	***	0.0008
P. aeruginosa vs. S. aureus	18.36	10.08 to 26.64	Yes	****	<0.0001
P. aeruginosa vs. E. faecalis	23.51	15.23 to 31.79	Yes	****	<0.0001
S. aureus vs. E. faecalis	5.144	-3.137 to 13.42	No	ns	0.3388
**++(Cu)**					
E. coli vs. P. aeruginosa	-10.00	-18.28 to -1.720	Yes	*	0.0138
E. coli vs. S. aureus	11.74	3.463 to 20.02	Yes	**	0.0034
E. coli vs. E. faecalis	-3.766	-12.05 to 4.514	No	ns	0.5994
P. aeruginosa vs. S. aureus	21.74	13.46 to 30.02	Yes	****	<0.0001
P. aeruginosa vs. E. faecalis	6.234	-2.047 to 14.51	No	ns	0.1893
S. aureus vs. E. faecalis	-15.51	-23.79 to -7.229	Yes	***	0.0002
**+++(Cu)**					
E. coli vs. P. aeruginosa	-9.000	-17.28 to -0.7197	Yes	*	0.0296
E. coli vs. S. aureus	5.000	-3.280 to 13.28	No	ns	0.3629
E. coli vs. E. faecalis	6.286	-1.995 to 14.57	No	ns	0.1836
P. aeruginosa vs. S. aureus	14.00	5.720 to 22.28	Yes	***	0.0005
P. aeruginosa vs. E. faecalis	15.29	7.005 to 23.57	Yes	***	0.0002
S. aureus vs. E. faecalis	1.286	-6.995 to 9.566	No	ns	0.9730
**Coated pegs – Biofilm on wells**					
**Tukey's multiple comparisons test**	**Mean Diff**	**95.00% CI of diff**	**Significant?**	**Summary**	**Adjusted P Value**
**+(Cu)**					
E. coli vs. P. aeruginosa	-7.764	-28.60 to 13.08	No	ns	0.7351
E. coli vs. S. aureus	-6.942	-27.78 to 13.90	No	ns	0.7950
E. coli vs. E. faecalis	-5.453	-26.29 to 15.39	No	ns	0.8874
P. aeruginosa vs. S. aureus	0.8220	-20.02 to 21.66	No	ns	0.9995
P. aeruginosa vs. E. faecalis	2.311	-18.53 to 23.15	No	ns	0.9898
S. aureus vs. E. faecalis	1.489	-19.35 to 22.33	No	ns	0.9972
**++(Cu)**					
E. coli vs. P. aeruginosa	3.961	-16.88 to 24.80	No	ns	0.9524
E. coli vs. S. aureus	10.83	-10.01 to 31.66	No	ns	0.4920
E. coli vs. E. faecalis	1.040	-19.80 to 21.88	No	ns	0.9990
P. aeruginosa vs. S. aureus	6.864	-13.98 to 27.70	No	ns	0.8004
P. aeruginosa vs. E. faecalis	-2.921	-23.76 to 17.92	No	ns	0.9799
S. aureus vs. E. faecalis	-9.785	-30.62 to 11.05	No	ns	0.5747
**+++(Cu)**					
E. coli vs. P. aeruginosa	-0.6129	-21.45 to 20.23	No	ns	0.9998
E. coli vs. S. aureus	3.482	-17.36 to 24.32	No	ns	0.9668
E. coli vs. E. faecalis	5.655	-15.19 to 26.49	No	ns	0.8764
P. aeruginosa vs. S. aureus	4.095	-16.74 to 24.93	No	ns	0.9478
P. aeruginosa vs. E. faecalis	6.267	-14.57 to 27.11	No	ns	0.8399
S. aureus vs. E. faecalis	2.173	-18.67 to 23.01	No	ns	0.9915
**Coated pegs – Biofilm on pegs**					
**Tukey's multiple comparisons test**	**Mean Diff**	**95.00% CI of diff**	**Significant?**	**Summary**	**Adjusted P Value**
**+(Cu)**					
E. coli vs. P. aeruginosa	11.57	-11.33 to 34.47	No	ns	0.5153
E. coli vs. S. aureus	12.04	-10.86 to 34.94	No	ns	0.4815
E. coli vs. E. faecalis	-1.016	-23.92 to 21.88	No	ns	0.9993
P. aeruginosa vs. S. aureus	0.4743	-22.42 to 23.37	No	ns	>0.9999
P. aeruginosa vs. E. faecalis	-12.58	-35.48 to 10.31	No	ns	0.4439
S. aureus vs. E. faecalis	-13.06	-35.96 to 9.840	No	ns	0.4120
**++(Cu)**					
E. coli vs. P. aeruginosa	11.94	-10.96 to 34.84	No	ns	0.4886
E. coli vs. S. aureus	23.13	0.2352 to 46.03	Yes	*	0.0471
E. coli vs. E. faecalis	8.016	-14.88 to 30.92	No	ns	0.7699
P. aeruginosa vs. S. aureus	11.19	-11.71 to 34.09	No	ns	0.5425
P. aeruginosa vs. E. faecalis	-3.926	-26.83 to 18.97	No	ns	0.9643
S. aureus vs. E. faecalis	-15.12	-38.02 to 7.781	No	ns	0.2883
**+++(Cu)**					
E. coli vs. P. aeruginosa	8.757	-14.14 to 31.66	No	ns	0.7194
E. coli vs. S. aureus	23.36	0.4616 to 46.26	Yes	*	0.0444
E. coli vs. E. faecalis	20.88	-2.021 to 43.78	No	ns	0.0829
P. aeruginosa vs. S. aureus	14.60	-8.295 to 37.50	No	ns	0.3168
P. aeruginosa vs. E. faecalis	12.12	-10.78 to 35.02	No	ns	0.4760
S. aureus vs. E. faecalis	-2.482	-25.38 to 20.42	No	ns	0.9905
